# High solar-to-hydrogen efficiency in a direct Z-scheme GaAs/GeH van der Waals heterostructure for photocatalytic water splitting

**DOI:** 10.1039/d6ra08869d

**Published:** 2026-10-16

**Authors:** Tran T. Nhan, Pham T. Truong, Le T. Ngoc Tu, Huynh V. Phuc, Chuong V. Nguyen, Ngoc-Trung Nguyen, Trang Minh Pham, Thi Kim Cuong Phu, Tam Duy Nguyen, Yoshiyuki Kawazoe, Thi Viet Bac Phung

**Affiliations:** a Faculty of Fundamental Sciences, Hanoi University of Industry 298 Cau Dien Hanoi 100000 Vietnam tran.nhan@haui.edu.vn; b Division of Physics, School of Education, Dong Thap University Cao Lanh 870000 Vietnam pttruong@dthu.edu.vn; c Department of Materials Science and Engineering, Le Quy Don Technical University Hanoi 100000 Vietnam; d Center for Environmental Intelligence, VinUniversity Hanoi 100000 Vietnam; e School of Chemistry, Monash University Melbourne Victoria 3800 Australia; f New Industry Creation Hatchery Center (NICHe), Tohoku University Sendai 980-8579 Japan; g Department of Centre for Inter Disciplinary Research, SRM University-AP Amaravati 522240 Andhra Pradesh India; h College of Engineering and Computer Science, VinUniversity Hanoi 100000 Vietnam bac.ptv@vinuni.edu.vn

## Abstract

The development of efficient photocatalysts for solar-driven water splitting remains a critical challenge due to limited light absorption, rapid charge recombination, and insufficient redox capability. In this work, we propose a GaAs/GeH van der Waals (vdW) heterostructure and systematically investigate its structural, electronic, mechanical, optical, and photocatalytic properties using first-principles calculations. The results demonstrate that the heterostructure is energetically, mechanically, and dynamically stable and exhibits a direct band gap with a type-II band alignment, enabling effective spatial separation of photogenerated charge carriers. The GaAs/GeH heterostructure shows a reduced band gap and significantly enhanced optical absorption in the visible region. In addition, improved electron mobility is observed, which is beneficial for charge transport. Importantly, the band-edge positions are well aligned with the redox potentials of water, allowing both hydrogen evolution reaction (HER) and oxygen evolution reaction (OER) to proceed. The system follows a direct Z-scheme charge transfer mechanism driven by an intrinsic built-in electric field, which preserves strong redox ability while suppressing carrier recombination. As a result, a theoretical solar-to-hydrogen (STH) conversion efficiency of 24.41% is achieved, demonstrating competitive photocatalytic performance compared with previously reported 2D heterostructures.These findings highlight the strong potential of the GaAs/GeH heterostructure for efficient solar-driven water splitting and provide valuable insights for the rational design of advanced photocatalytic materials.

## Abstract 2

The development of efficient photocatalysts for solar-driven water splitting remains a critical challenge due to limited light absorption, rapid charge recombination, and insufficient redox capability.

## Graphical abstract

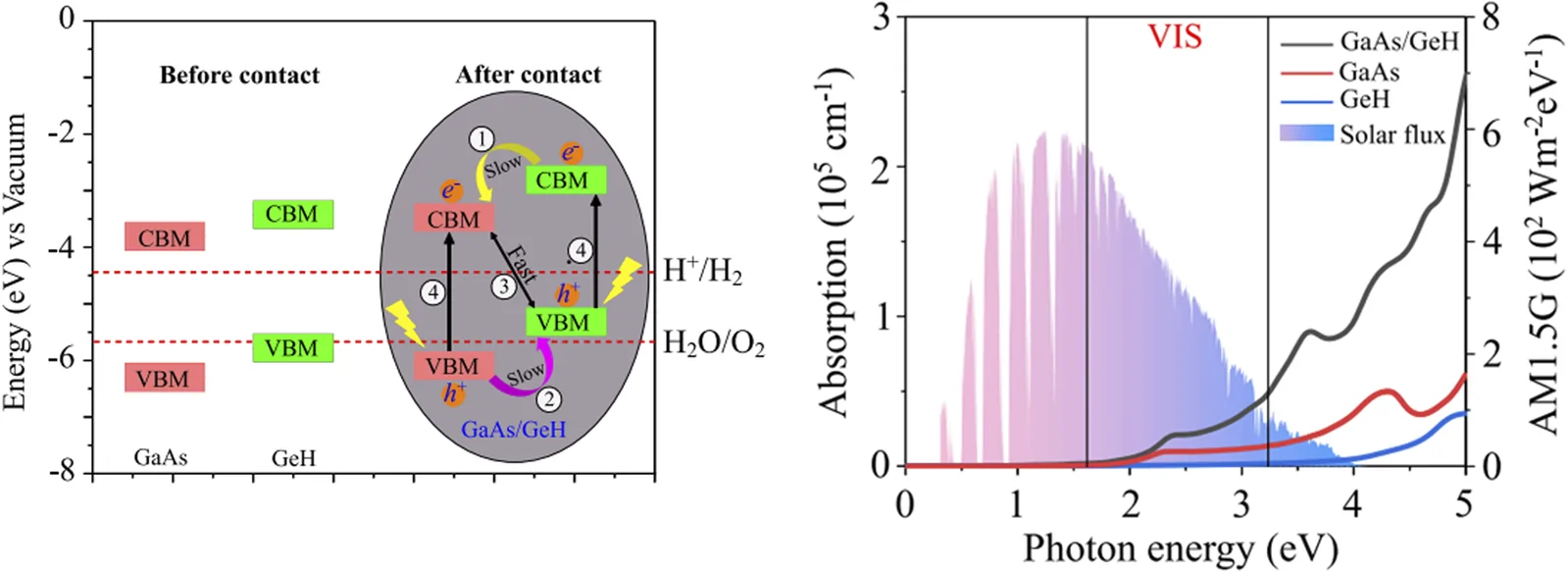

## 1 Introduction

The rapid increase in global energy consumption, together with escalating environmental concerns, has intensified the search for sustainable energy conversion technologies.^1–3^ Among the proposed solutions, hydrogen production *via* solar-driven water splitting is widely considered a viable route owing to its high gravimetric energy density and environmentally friendly nature.^4,5^ However, the overall efficiency of photocatalytic systems remains limited by several fundamental factors, including insufficient solar absorption, rapid recombination of photogenerated charge carriers, and the mismatch between band edge positions and the required redox potentials.^6–8^ The emergence of two-dimensional (2D) materials provides a flexible platform to address these challenges.^9,10^ Since the experimental isolation of graphene in 2004,^11^ low-dimensional systems have been extensively explored due to their pronounced quantum confinement effects and high surface sensitivity. These characteristics enable effective modulation of electronic structures and facilitate applications in both catalytic and optoelectronic processes. Following this development, a broad family of 2D materials, including transition-metal dichalcogenides,^12^ black phosphorus,^13^ and group IV–VI compounds,^14^ has been systematically investigated, revealing diverse electronic and optical properties. Despite these advances, individual monolayers often exhibit intrinsic trade-offs among light absorption, carrier transport, and redox activity, which limit their standalone performance.^15^

To overcome these limitations, the construction of vdW heterostructures *via* vertical stacking of distinct monolayers has emerged as an effective strategy.^16–18^ In contrast to conventional heterostructures, such systems are not constrained by strict lattice matching, thereby allowing a wider range of material combinations. More importantly, interfacial interactions can induce charge redistribution and generate an internal electric field, which plays a crucial role in regulating carrier separation and transfer dynamics. Depending on the resulting band alignment, these heterostructures may either promote spatial separation of electrons and holes or preserve strong redox capability, both of which are advantageous for photocatalytic applications.^19–22^ Type-II band alignment is widely regarded as an effective mechanism for enhancing charge separation in photocatalytic systems.^23–28^ In this configuration, the conduction band minimum (CBM) and valence band maximum (VBM) are located in different constituent layers, enabling photogenerated electrons and holes to migrate in opposite directions across the interface. This spatial separation suppresses recombination and prolongs carrier lifetime, thereby improving photocatalytic efficiency.

Among the various candidates, gallium arsenide (GaAs), a representative group III–V two-dimensional (2D) material, has attracted increasing interest due to its favorable electronic structure and strong light–matter interaction, making it suitable for optoelectronic and photocatalytic applications.^29,30^ Both theoretical and experimental studies have demonstrated its strong optical absorption, suitable band edge alignment for water splitting, and high carrier mobility.^31,32^ In addition, monolayer GaAs has been identified as a promising component for constructing vdW heterostructures with other 2D materials, offering enhanced photocatalytic performance through interfacial electronic modulation.^33–37^ On the other hand, hydrogenated germanene (GeH) has emerged as a stable derivative of germanene with a modified bonding configuration.^38,39^ Compared to pristine germanene, the hydrogenation process not only enhances structural stability but also opens a band gap, transforming it into a semiconducting material with potential applications in nanoelectronics and energy conversion.^40^ In addition, GeH exhibits relatively high carrier mobility and tunable electronic properties, which are advantageous for facilitating charge transport.^41^ The GeH-based heterostructures for photocatalytic water splitting have also been explored, including GeH/GeCH_3_ (ref. 42) and GeH/InSe.^42–44^

Given the extraordinary properties of GaAs and GeH, their combination into vdW heterostructure is expected to provide a promising platform for efficient photocatalytic water splitting. Motivated by these considerations, in this work, we systematically investigate the structural stability, electronic structure, carrier transport, and optical properties of the GaAs/GeH vdW heterostructure using first-principles calculations. Special attention is devoted to understanding the interfacial charge transfer mechanism, band alignment characteristics, and their implications for photocatalytic water splitting. Furthermore, the solar-to-hydrogen (STH) conversion efficiency is evaluated to assess the practical potential of the proposed system. Our findings provide valuable insights into the design of high-performance 2D heterostructures for sustainable energy applications.

## 2 Computational methods

All calculations were performed within the framework of density functional theory (DFT)^45^ as implemented in the Quantum ESPRESSO package.^46,47^ The interaction between ions and valence electrons was described using the projector augmented wave (PAW) method,^48^ while the exchange–correlation effects were treated within the generalized gradient approximation (GGA)^49^ in the form of the Perdew–Burke–Ernzerhof (PBE) functional.^50^ A plane-wave basis set with a kinetic energy cutoff of 500 eV was employed to ensure convergence of total energy. The Brillouin zone was sampled using a Monkhorst–Pack *k*-point mesh of 15 × 15 × 1 for structural relaxation and electronic structure calculations. All atomic positions were fully optimized until the residual forces on each atom were less than 10^−3^ eV Å^−1^, and the total energy convergence criterion was set to 10^−6^ eV. To avoid spurious interactions between periodic images, a vacuum layer of at least 25 Å was introduced along the direction perpendicular to the monolayer plane. The vdW interaction between layers in the heterostructure was taken into account using the DFT-D3 correction scheme proposed by Grimme.^51^ In addition, the Heyd–Scuseria–Enzerhof (HSE06) hybrid functional^52^ was employed to obtain more accurate band gap values.

## 3 Results and discussion

### 3.1 Electronic properties of GaAs and GeH monolayers

We first examine the atomic structures, electronic properties, and stability of the GaAs and GeH monolayers, as shown in Fig. 1. The GaAs monolayer adopts a buckled honeycomb structure, in which Ga and As atoms are arranged in a hexagonal lattice, as depicted in Fig. 1(a). Each Ga atom is bonded to three neighboring As atoms, while each As atom is coordinated with three Ga atoms, forming a typical hexagonal network. The buckling height between the Ga and As sublayers is calculated to be 0.59 Å. In addition, the optimized lattice constant is 4.03 Å. These values are consistent with previously reported results.^30,34^ Similarly, the GeH monolayer adopts a buckled structure associated with the sp^3^ hybridization of Ge atoms. Hydrogen atoms are alternately bonded on both sides of the layer, as illustrated in Fig. 1(b). The buckling height between the two Ge sublayers is calculated to be 0.79 Å, which is larger than that of the GaAs monolayer. The optimized lattice constant is 4.04 Å. These values are consistent with previously reported results,^43,53^ supporting the validity of our computational approach.

**Fig. 1:**
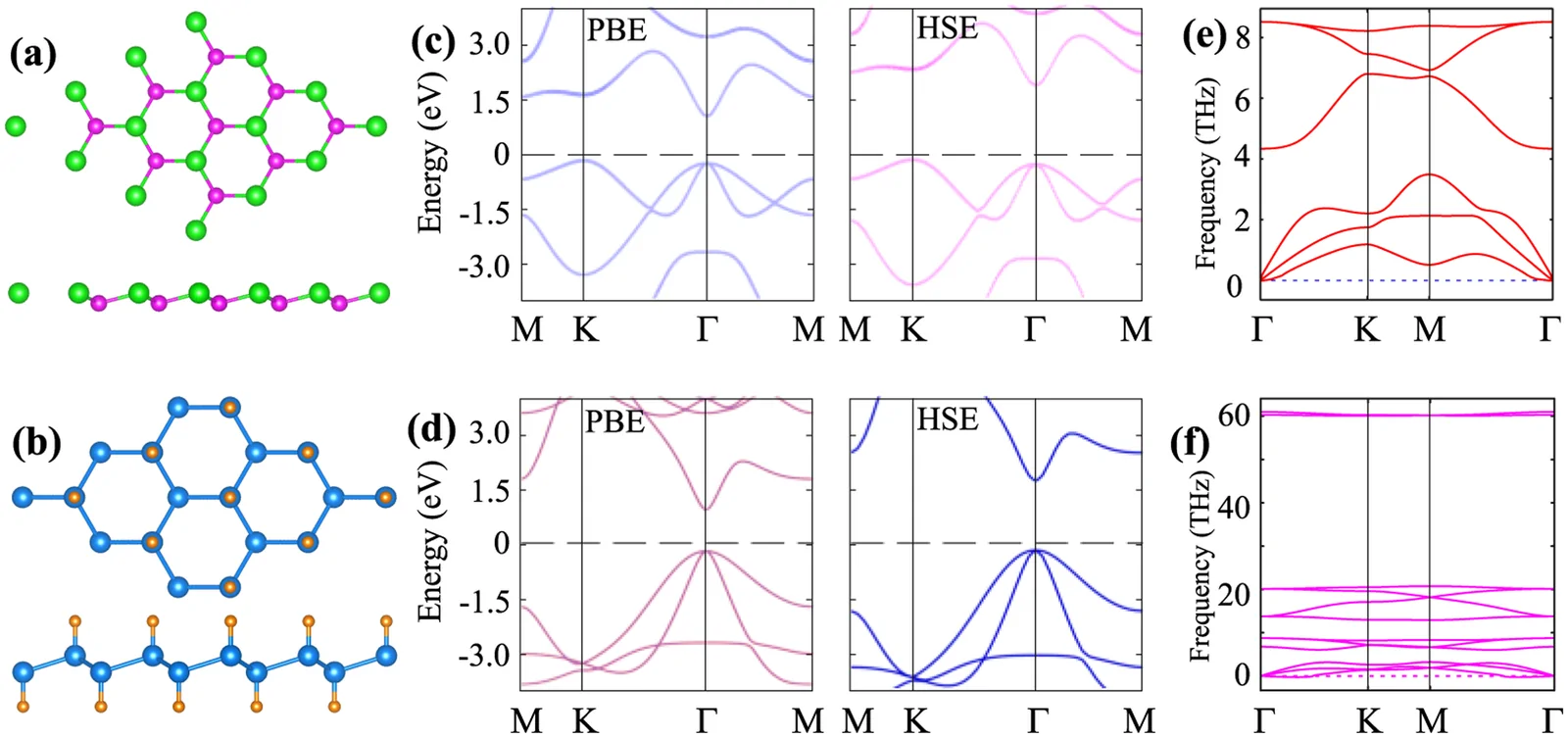
Atomic structure of (a) GaAs and (b) GeH monolayers, band structures of (c) GaAs and (d) GeH monolayers, phonon dispersion curves of (e) GaAs and (f) GeH monolayers.

Furthermore, the electronic band structures of the GaAs and GeH monolayers are shown in Fig. 1(c) and (d), respectively. Spin–orbit coupling (SOC) was not explicitly considered in the initial calculations, as its effect on the electronic properties of GaAs monolayer has been reported to be relatively small.^54^ The GaAs monolayer exhibits semiconducting behavior with an indirect band gap, where the VBM and CBM are located at the K and Γ points, respectively. The calculated band gap is 1.21 eV using the PBE functional and increases to 2.02 eV when the HSE06 functional is employed. In contrast, the GeH monolayer is a direct band gap semiconductor, with both the VBM and CBM located at the Γ point. The corresponding band gaps are 1.13 eV and 1.88 eV as obtained from the PBE and HSE06 calculations, respectively. A comparison between the two methods indicates that the main difference lies in the upward shift of the CBM when using the HSE06 functional, leading to an enlarged band gap. Despite this quantitative difference, both approaches consistently predict the same qualitative electronic characteristics for the two monolayers. Moreover, to further evaluate their dynamical stability, phonon dispersion calculations were performed, as displayed in Fig. 1(e) and (f). The absence of imaginary frequencies throughout the Brillouin zone confirms that both GaAs and GeH monolayers are dynamically stable.

### 3.2 Atomic structures of GaAs/GeH vdW heterostructure

Based on the optimized geometries of the individual monolayers, the GaAs/GeH heterostructure is constructed by vertically stacking the two layers. Various stacking configurations are considered to identify the most stable arrangement, as illustrated in Fig. 2. These configurations are generated by varying the relative positions between the layers, including high-symmetry arrangements in which Ga (or As) atoms are aligned with Ge or H atoms. For each configuration, the interlayer distance is fully relaxed to obtain the equilibrium structure. The corresponding structural and electronic parameters, including interlayer distance, binding energy, and band gap values, are summarized in Table 1. It is observed that the interlayer distance varies significantly among different stacking configurations. The largest interlayer distance is found in the Stack@AB1 configuration (2.98 Å), while the smallest interlayer distance is obtained for the Stack@AC2 configuration (2.10 Å). Moreover, the calculated interlayer distances are comparable to those reported for other typical vdW systems, such as SiH-based^55,56^ and GeH-based structures.^57,58^ This similarity indicates that the interlayer interactions in the GaAs/GeH system are primarily governed by weak vdW forces.

**Fig. 2:**
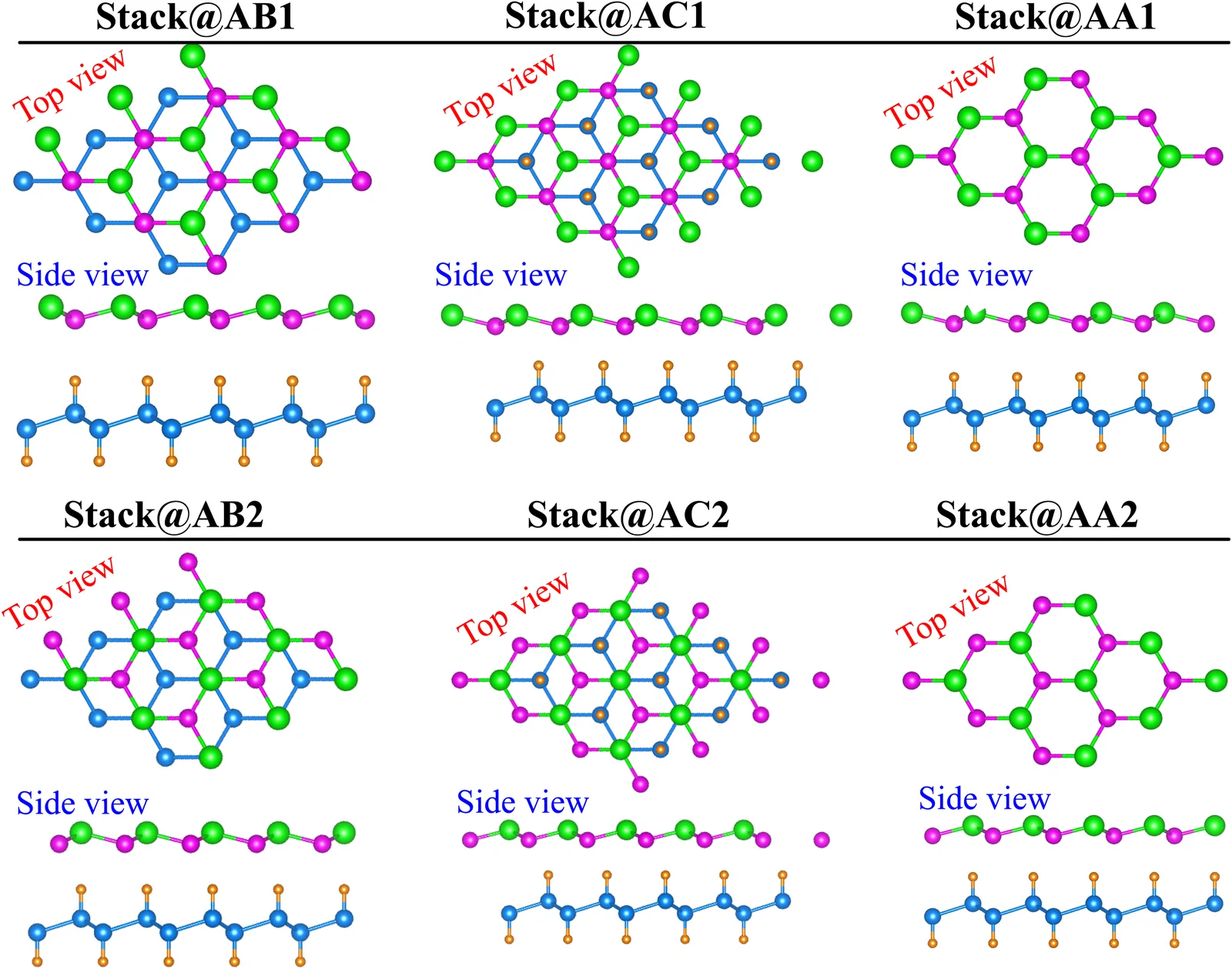
Atomic structures of the GaAs/GeH heterostructure with different stacking configurations.

**Table 1:** Calculated interlayer distance (*d*, Å), binding energy (*E*_b_, meV Å^−2^), band gap obtained by PBE and HSE functional (eV) of the GaAs/GeH heterostructure for different stacking configurations

Stackings	*d*	*E*_b_	*E* ^PBE^_g_	*E* ^HSE^_g_	Behavior	Contact types
Stack@AB1	2.98	−8.69	0.50	1.20	Direct	Type-II
Stack@AC1	2.63	−11.56	0.56	1.29	Direct	Type-II
Stack@AA1	2.95	−8.77	0.50	1.20	Direct	Type-II
Stack@AB2	2.42	−11.99	0.66	1.39	Direct	Type-II
Stack@AC2	2.10	−14.82	0.65	1.37	Direct	Type-II
Stack@AA2	2.37	−12.21	0.68	1.41	Direct	Type-II

Furthermore, to evaluate the stability of the GaAs/GeH heterostructure, the binding energy is calculated as:
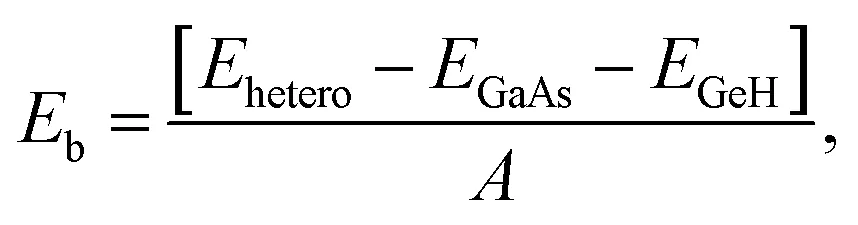
where *E*_hetero_, *E*_GaAs_, and *E*_GeH_ denote the total energies of the heterostructure, isolated GaAs, and GeH monolayers, respectively, and *A* represents the surface area of the heterostructure. A negative binding energy indicates that the formation of the heterostructure is energetically favorable. As shown in Table 1, the Stack@AC2 configuration exhibits the lowest binding energy among all considered structures. Combined with its shortest interlayer distance, this result indicates that Stack@AC2 is the most energetically stable configuration.

### 3.3 Electronic properties and contact behavior of the GaAs/GeH heterostructure

To gain deeper insight into the electronic properties of the GaAs/GeH heterostructure, the projected band structures for different stacking configurations are presented in Fig. 3. The contributions from the GaAs and GeH layers are distinguished by red and blue lines, respectively. All considered configurations exhibit semiconducting behavior with direct band gaps. The projected bands indicate that the intrinsic electronic characteristics of the individual layers are largely retained in the heterostructure, which can be attributed to the weak vdW interaction between them. In particular, the GaAs component preserves its indirect band feature, while the GeH layer maintains its direct band edge character. More importantly, the projected band structures reveal a typical type-II band alignment, in which the VBM is primarily contributed by the GeH layer, whereas the CBM originates from the GaAs layer. This spatial separation of charge carriers across the interface is beneficial for suppressing electron–hole recombination and enhancing carrier lifetime. Such a type-II band alignment is highly desirable for photocatalytic applications, as it promotes directional charge transfer and enhances carrier lifetime. These results suggest that the GaAs/GeH heterostructure is a promising candidate for photocatalytic water splitting. In addition, the formation of type-II band alignment in the GaAs/GeH heterostructure leads to a reduced band gap compared to those of the constituent monolayers, as summarized in Table 1. This reduction can be attributed to the staggered band alignment at the interface, which effectively narrows the energy difference between the band edges. As a result, the GaAs/GeH heterostructure is expected to exhibit enhanced optical absorption in the visible region. This feature is particularly advantageous for photocatalytic applications, as it allows more efficient utilization of the solar spectrum.

**Fig. 3:**
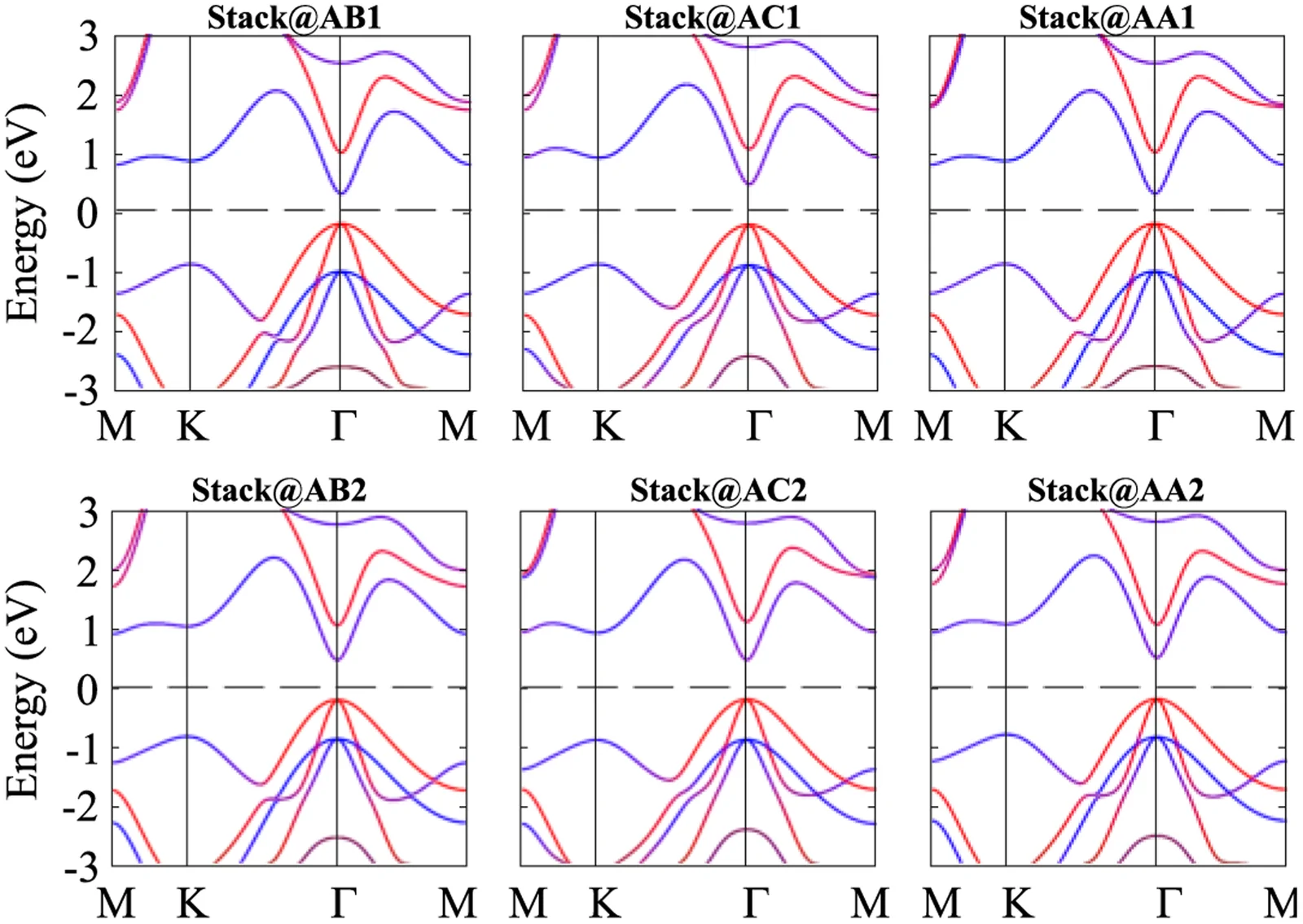
Projected band structures of the GaAs/GeH heterostructure with different stacking configurations. Red and blue lines represent the contributions of the GaAs and GeH layers, respectively.

To further elucidate the electronic characteristics of the GaAs/GeH heterostructure, the projected density of states (PDOS) for different stacking configurations is presented in Fig. 4. It can be clearly observed that the states near the VBM of the GaAs/GeH heterostructure for all stacking configurations are predominantly contributed by the Ge p orbital of the GeH layer, whereas the CBM is mainly derived from the strong hybridization between Ga and As orbitals in the GaAs layer. This distribution of electronic states further verifies the formation of a type-II band alignment in the GaAs/GeH heterostructure. These findings are in good agreement with the band structure analysis and further confirm that the GaAs/GeH heterostructure is favorable for applications requiring efficient charge separation, such as photocatalytic water splitting.

**Fig. 4:**
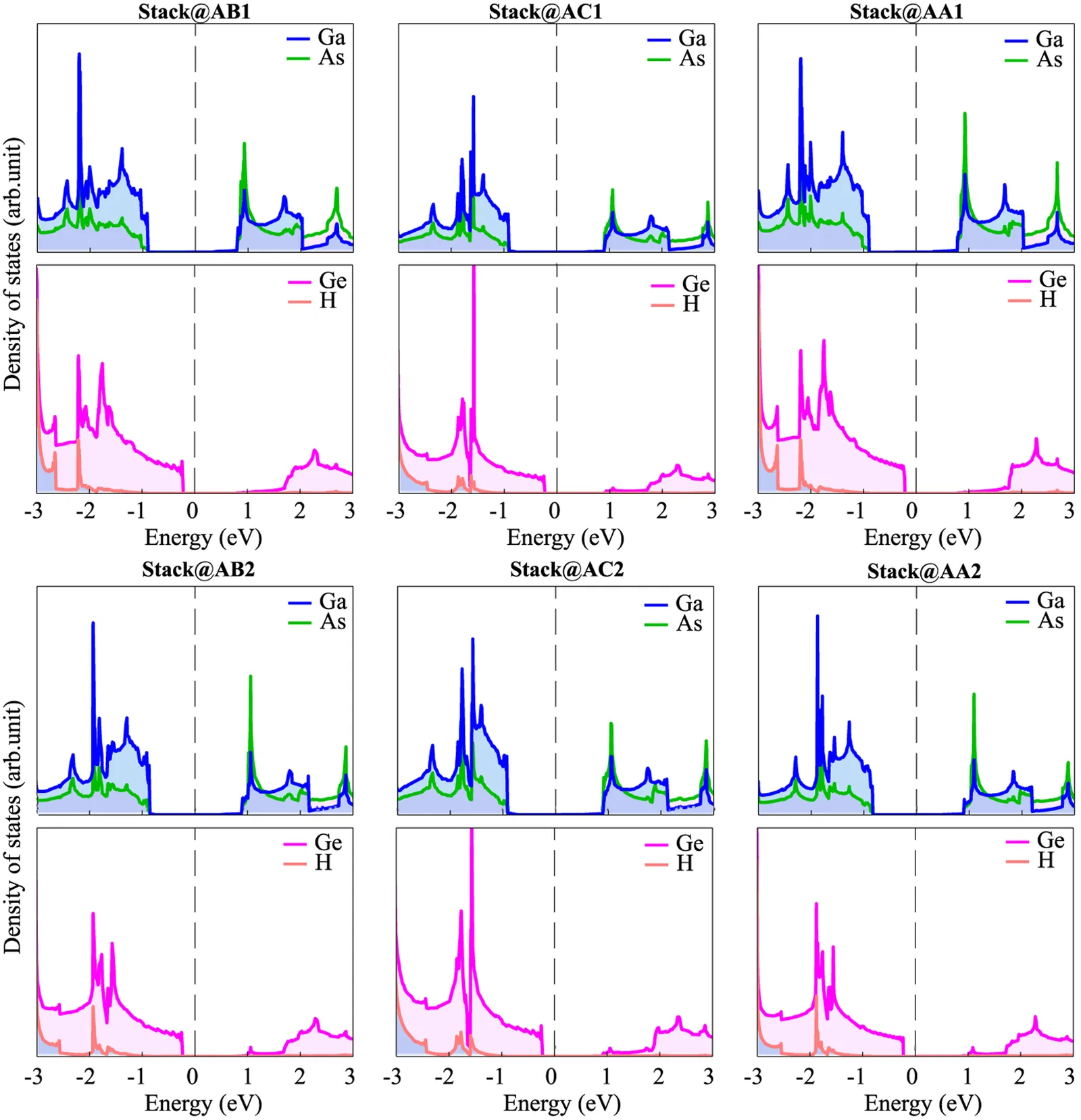
Projected density of states (PDOS) of the GaAs/GeH heterostructure with different stacking configurations.

To further validate the electronic properties, the projected band structures calculated using the HSE06 functional are presented in Fig. 5. Compared to the PBE results, the overall band dispersion remains largely unchanged, while the band gaps are significantly increased due to the more accurate treatment of exchange–correlation effects, as listed in Table 1. The upward shift of the conduction bands is primarily responsible for the band gap enlargement in the HSE06 calculations. Importantly, the type-II band alignment is well preserved across all stacking configurations. The VBM remains predominantly contributed by the GeH layer, whereas the CBM is mainly derived from the GaAs layer, consistent with the PBE results. This indicates that the spatial separation of electrons and holes is robust and does not depend on the choice of exchange–correlation functional. The increase in band gap values obtained from the HSE06 functional suggests improved reliability in describing the electronic structure, particularly for evaluating photocatalytic performance.

**Fig. 5:**
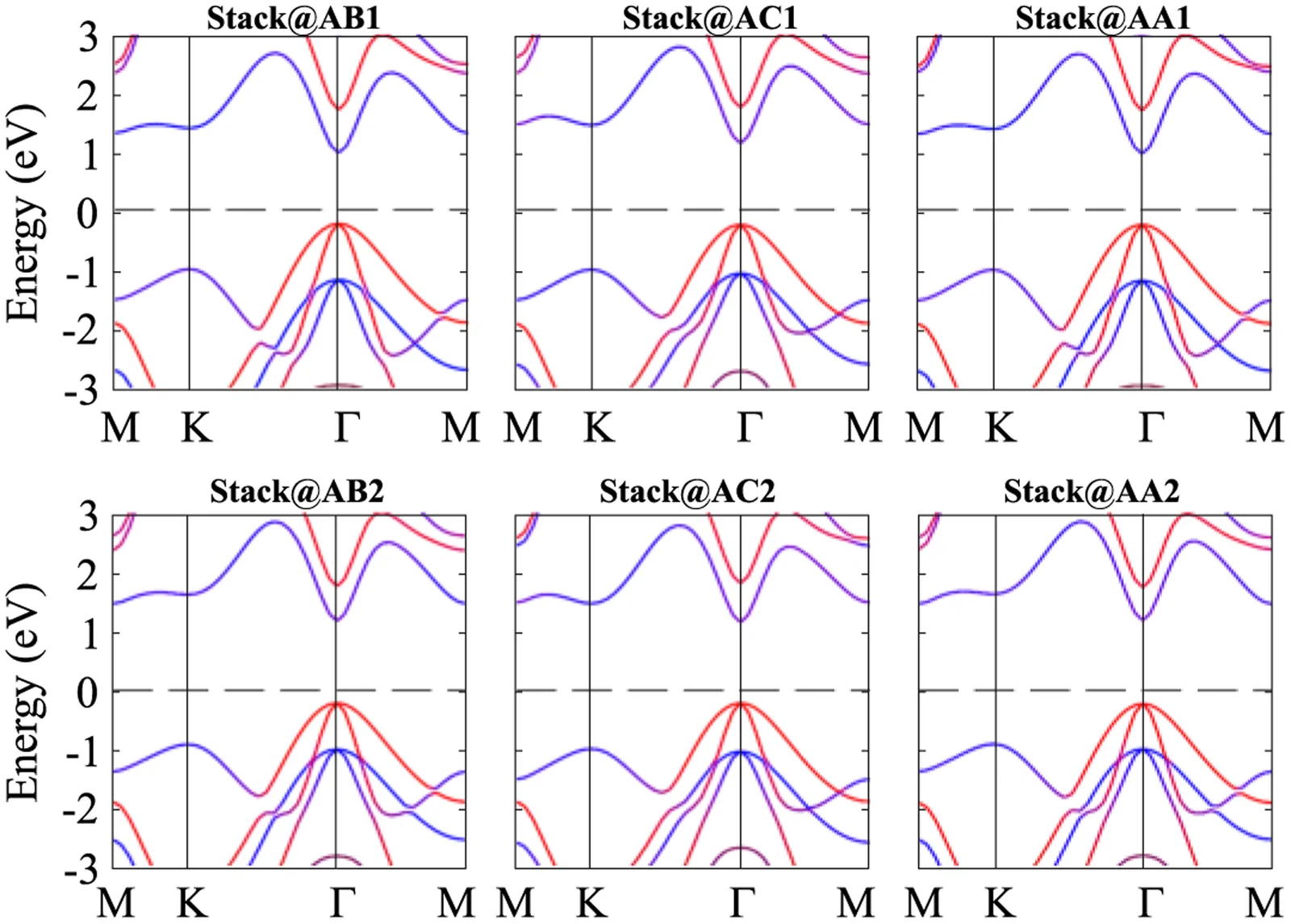
Projected band structures of the GaAs/GeH heterostructure with different stacking configurations obtained from HSE06 functional. Red and blue lines represent the contributions of the GaAs and GeH layers, respectively.

### 3.4 The stability of the GaAs/GeH heterostructure

As discussed above, the Stack@AC2 configuration is identified as the most energetically stable structure. Therefore, we focus on this configuration to further investigate its mechanical and dynamical stability, as well as its potential for electronic and photocatalytic water splitting applications. First, the elastic constants are calculated to evaluate the mechanical properties of the GaAs/GeH heterostructure, as illustrated in Fig. 6(a). For a 2D hexagonal system, the in-plane elastic behavior is described by four independent constants, namely *C*_11_ = *C*_22_, *C*_12_, and *C*_66_. The calculated *C*_11_ of the GaAs/GeH heterostructure is 97 N m^−1^, which is significantly higher than those of the isolated GaAs (50 N m^−1^) and GeH (48 N m^−1^) monolayers. Similarly, the value of *C*_12_ is 26 N m^−1^, exceeding those of GaAs (16 N m^−1^) and GeH (10 N m^−1^), while a comparable trend is also observed for *C*_66_. These results indicate that the formation of the GaAs/GeH heterostructure enhances the in-plane stiffness compared to the individual monolayers. Furthermore, the calculated elastic constants satisfy the Born stability criteria, *i.e.*, *C*_11_^2^ − *C*_12_^2^ > 0 and *C*_66_ > 0, confirming the mechanical stability of the heterostructure. Furthermore, the angular dependence of the Young's modulus, which can be expressed in terms of the elastic constants *C*_*ij*_ as follows:1
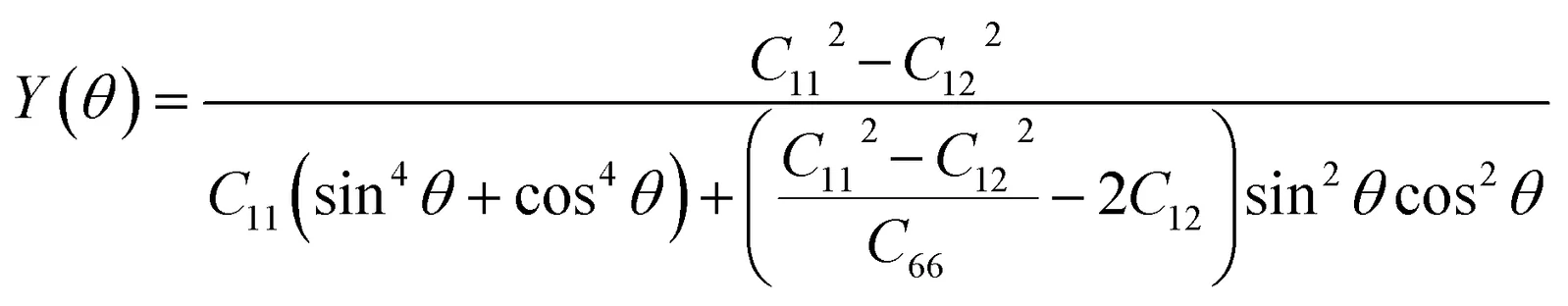


**Fig. 6:**
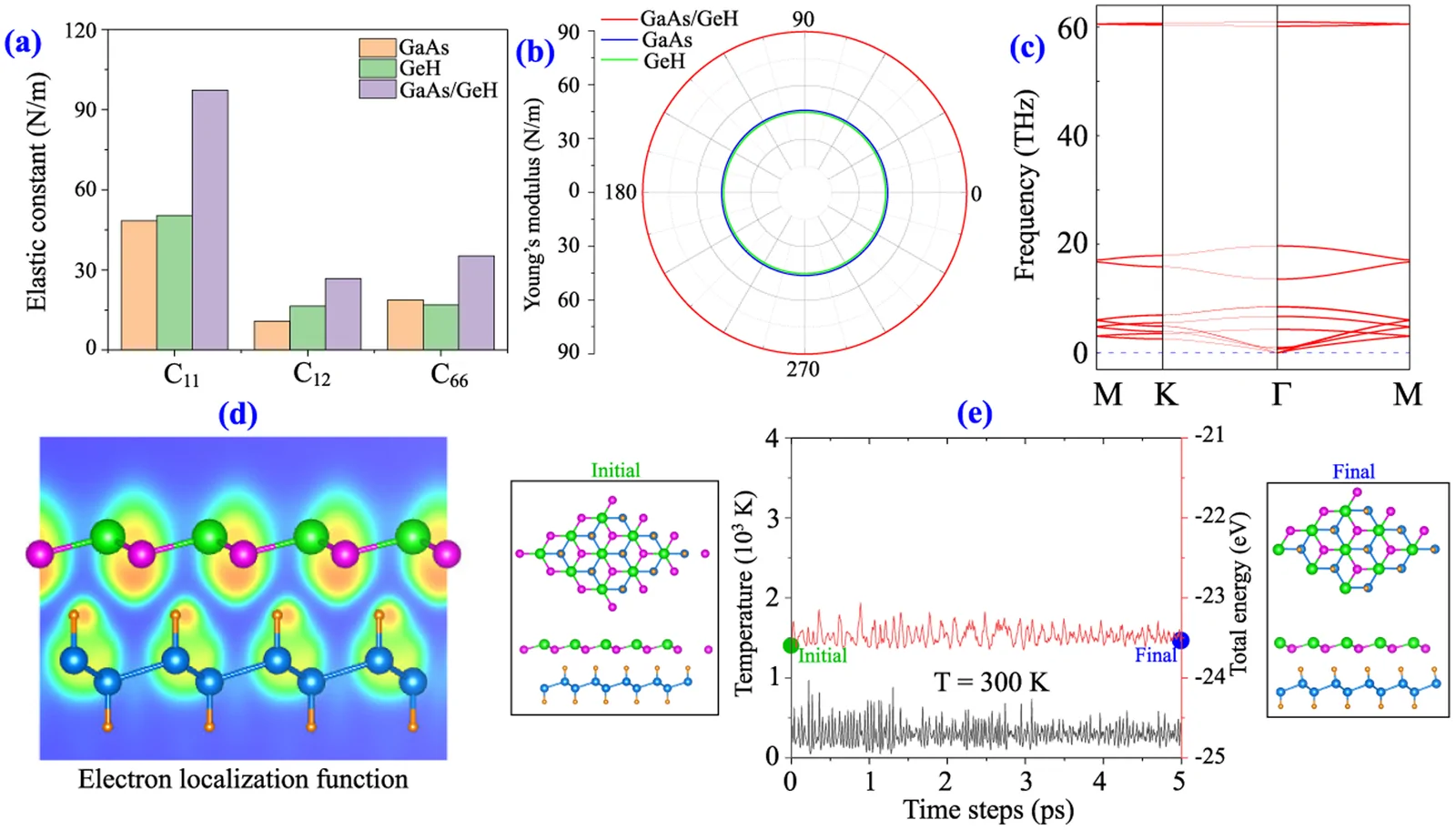
(a) Elastic constants, (b) Young's modulus, (c) phonon spectrum, (d) electron localization function (ELF) and (e) AIMD simulations of the GaAs/GeH heterostructure.

The angular dependence of the Young's modulus for the GaAs/GeH heterostructure is shown in Fig. 6(b). The polar plot exhibits a circular shape, indicating that the heterostructure possesses isotropic in-plane mechanical properties. In addition, the Young's modulus of the GaAs/GeH heterostructure is calculated to be 90 N m^−1^, which is significantly higher than those of the GeH (46 N m^−1^) and GaAs (45 N m^−1^) monolayers. This enhancement indicates that the formation of the heterostructure improves the in-plane stiffness, likely due to the interlayer coupling between the two constituent layers. Such an increase in stiffness is beneficial for maintaining structural integrity under external mechanical perturbations.

Furthermore, the dynamical stability of the GaAs/GeH heterostructure is verified by analyzing the phonon spectrum, as depicted in Fig. 6(c). No imaginary frequencies are observed throughout the Brillouin zone, indicating that the heterostructure is dynamically stable. This result suggests that the GaAs/GeH heterostructure can maintain its stability under small perturbations and is feasible for experimental realization. To gain further insight into the interfacial interaction, the electron localization function (ELF) of the GaAs/GeH heterostructure is examined, as presented in Fig. 6(d). The ELF distribution reveals pronounced electron localization within the GaAs and GeH layers, indicating that the intralayer bonding retains its covalent character. In contrast, the region between the two layers exhibits a much lower degree of electron localization, suggesting minimal orbital overlap across the interface. This behavior implies that no significant chemical bonding is formed between the layers. Such a spatial distribution of electron density supports the conclusion that the two monolayers are coupled predominantly through non-covalent interactions. This weak interlayer coupling is advantageous for maintaining the intrinsic electronic features of each component while still allowing interfacial charge transfer. Furthermore, the thermal stability of the GaAs/GeH heterostructure was further assessed by performing an *ab initio* molecular dynamics (AIMD) simulation at 300 K, as shown in Fig. 6(e). During the simulation, the total energy fluctuates around an equilibrium value without any significant variation, indicating that the heterostructure maintains its structural integrity at room temperature. No apparent structural reconstruction and bond breaking is observed throughout the simulation. These results confirm the thermal stability of the GaAs/GeH heterostructure.

### 3.5 Charge transfer mechanism in the GaAs/GeH heterostructure

Furthermore, to gain deeper insight into the charge transfer mechanism at the GaAs/GeH interface, the work functions of the isolated GeH and GaAs monolayers, as well as their combined heterostructure, are analyzed, as shown in Fig. 7(a–c). The work function is defined as:

2 *Φ* = *E*_vac_ − *E*_F_

where *E*_vac_ and *E*_F_ represent the vacuum level and Fermi level, respectively. As depicted in Fig. 7, the work functions of the GeH monolayer are calculated to be 5.54 eV for both surfaces, indicating its out-of-plane symmetry. In contrast, the GaAs monolayer exhibits asymmetric work functions of 6.02 and 5.32 eV for its two surfaces, reflecting the broken out-of-plane symmetry. Upon forming the heterostructure, the work functions of the GaAs/GeH system are found to be 5.54 and 4.45 eV for the two outer surfaces, as shown in Fig. 7(c). This change indicates a significant redistribution of the electrostatic potential induced by the interfacial interaction between the two layers. Furthermore, the difference in surface work functions provides important information about the direction of interfacial charge redistribution. In the relaxed GaAs/GeH heterostructure, the GeH layer directly contacts the As-terminated surface of GaAs, which corresponds to the GaAs surface with a work function of 6.02 eV. Therefore, the relevant work-function difference at the interface is *Φ*_GeH_ = 5.54 eV < *Φ*_As-terminatedGaAs_ = 6.02 eV. This work-function difference indicates that electrons preferentially transfer from the GeH layer to the GaAs layer upon contact until Fermi-level equilibration is achieved. Consequently, the GeH layer becomes relatively electron depleted, whereas the GaAs layer gains electron density near the interface, resulting in the formation of an interfacial dipole and an intrinsic electric field across the heterostructure. The resulting potential difference between the two outer surfaces of the heterostructure is Δ*Φ* = 1.09 eV, as shown in Fig. 7(c). The associated intrinsic electric field (*E*_in_) is directed from the relatively electron-depleted GeH side toward the relatively electron-accumulated GaAs side. This built-in electric field provides an electrostatic driving force that influences the migration of photogenerated carriers and contributes to their spatial separation.

**Fig. 7:**
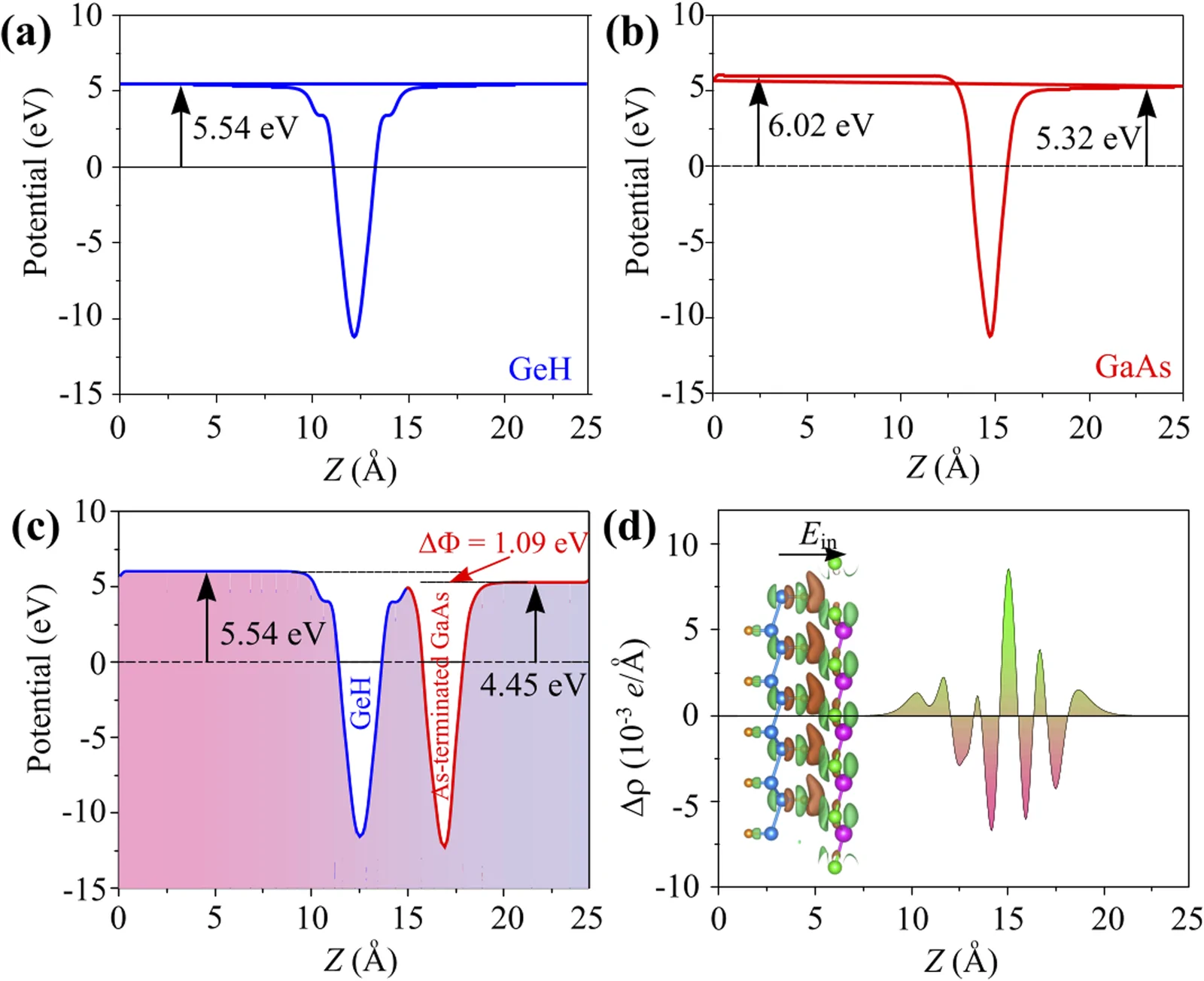
The average electrostatic potentials of (a) GeH, (b) GaAs monolayers and (c) GaAs/GeH heterostructure. (d) The planar average charge density difference of the GaAs/GeH heterostructure. The inset visualizes 3D isosurface of the GaAs/GeH heterostructure with the charge accumulation (green) and depletion (orange).

To further visualize the interfacial charge redistribution, the charge density difference is presented in Fig. 7(d), and is defined as follows:

3 Δ*ρ* = *ρ*_GaAs/GeH_ − *ρ*_GaAs_ − *ρ*_GeH_

where *ρ*_GaAs/GeH_, *ρ*_GaAs_, and *ρ*_GeH_ denote the charge densities of the heterostructure, isolated GaAs, and GeH monolayers, respectively. As shown in Fig. 7(d), electron depletion is mainly observed in the GeH region, whereas electron accumulation occurs around the GaAs layer. This charge redistribution indicates that electrons are transferred from the GeH layer to the GaAs layer upon formation of the heterostructure. To quantitatively determine the direction and magnitude of the interfacial charge transfer, Bader charge analysis was further performed for the GaAs/GeH heterostructure. The calculated net charge transfer is approximately 1.45 × 10^−2^*e* from the GeH layer to the GaAs layer, indicating electron depletion in GeH and electron accumulation in GaAs layer. This result is fully consistent with the work-function difference between GeH (5.54 eV) and the As-terminated GaAs surface (6.02 eV), which drives electron transfer from GeH toward GaAs upon heterostructure formation.

### 3.6 Carrier mobility of the GaAs/GeH heterostructure

Building upon the above analysis of electronic structure and interfacial charge transfer, it is essential to further evaluate the charge transport characteristics of the GaAs/GeH heterostructure. The carrier mobility is calculated based on the deformation potential (DP) theory proposed by Bardeen and Shockley.^59^ Within this framework, the carrier mobility in two-dimensional materials can be expressed as:4
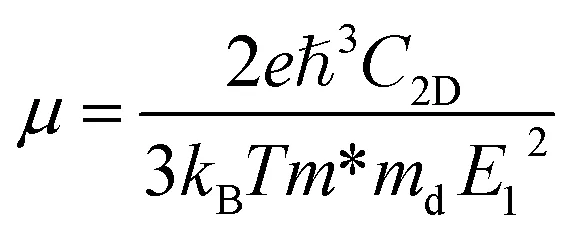
where *e* is the elementary charge, ℏ is the reduced Planck constant, *k*_B_ is the Boltzmann constant, and *T* is the temperature (taken as 300 K). m* is the effective mass of the carrier along the transport direction, while *m*_d_ is the average effective mass defined as 
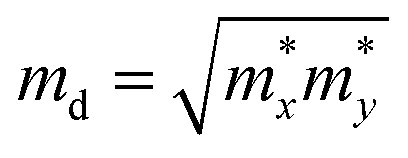
. *C*_2D_ represents the in-plane stiffness of the material, and *E*_1_ is the deformation potential constant, which characterizes the shift of the band edge under small strain. The carrier mobility of the GaAs, GeH monolayers and the GaAs/GeH heterostructure along the *x* and *y* directions is summarized in Table 2. For the GaAs monolayer, the hole mobility is significantly higher than the electron mobility in both directions, reaching values of 3464 and 5085 cm^2^ V^−1^ s^−1^ along the *x* and *y* directions, respectively. For the GeH monolayer, the electron mobility dominates, with values of 1583 and 1784 cm^2^ V^−1^ s^−1^ along the *x* and *y* directions, respectively, whereas holes exhibit much lower mobility (around 526 cm^2^ V^−1^ s^−1^). Interestingly, upon forming the GaAs/GeH heterostructure, the electron mobility is significantly enhanced, reaching 2352 and 2858 cm^2^ V^−1^ s^−1^ along the *x* and *y* directions, respectively. These values are comparable with those obtained in other 2D vdW heterostructures, such as GaAs/Bi_2_Se_3_,^60^ GaSe/ZrS_2_ (ref. 61) and GeH/InSe.^43^ On the other hand, the hole mobility in the heterostructure is reduced to approximately 482–519 cm^2^ V^−1^ s^−1^, which is much lower than that of the GaAs monolayer. All these findings suggest that the GaAs/GeH heterostructure exhibits a pronounced electron-dominated transport behavior with high electron mobility. This characteristic is highly favorable for photocatalytic applications, as it facilitates efficient electron transport while reducing the recombination probability of charge carriers.

**Table 2:** Calculated 2D elastic modulus (*C*_2D_), deformation potential constant (*E*_1_), effective mass (m*), and carrier mobility (*µ*_2D_) of GaAs, GeH, and GaAs/GeH heterostructure along the *x* and *y* directions

Materials	Directions	Carriers	*C*_2D_ (N m^−1^)	*E*_1_ (eV)	m* (*m*_e_)	*µ*_2D_ (cm^2^ V^−1^ s^−1^)
GaAs	*x*	Electron	58.27	−10.64	0.094	1240
	*x*	Hole	58.27	−0.95	0.630	3464
	*y*	Electron	58.27	−10.64	0.101	1074
	*y*	Hole	58.27	−0.95	0.520	5085
GeH	*x*	Electron	59.11	−10.37	0.086	1583
	*x*	Hole	59.11	−3.10	0.500	524
	*y*	Electron	59.11	−10.37	0.081	1784
	*y*	Hole	59.11	−3.10	0.499	526
GaAs/GeH	*x*	Electron	118.58	−11.91	0.087	2352
	*x*	Hole	118.58	−4.41	0.519	482
	*y*	Electron	118.58	−11.90	0.079	2858
	*y*	Hole	118.58	−4.41	0.500	519

### 3.7 Optical properties of the GaAs/GeH heterostructure

To further assess the suitability of the GaAs/GeH heterostructure for photocatalytic applications, it is essential to investigate its optical absorption properties, which determine the efficiency of solar energy harvesting. To evaluate the optical response of the GaAs, GeH monolayers and the GaAs/GeH heterostructure, the frequency-dependent dielectric function is calculated. The optical absorption coefficient can be obtained from the real and imaginary parts of the dielectric function using the following relation:5
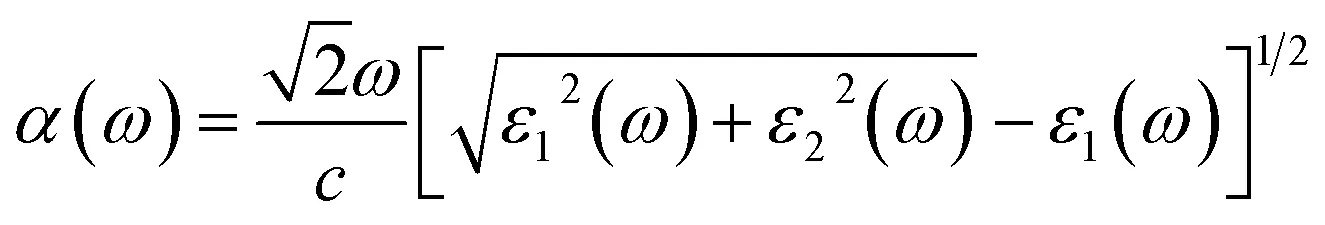
where *ω* is the photon frequency, *c* is the speed of light, and *ε*_1_(*ω*) and *ε*_2_(*ω*) are the real and imaginary parts of the dielectric function, respectively. The calculated absorption spectra are presented in Fig. 8. Notably, the GaAs/GeH heterostructure exhibits a pronounced enhancement in optical absorption compared to the individual monolayers, particularly in the visible and ultraviolet regions. This improvement is primarily attributed to the reduced band gap and the synergistic interaction between the two constituent layers, which extends the absorption range and enhances optical transition probabilities. Compared with the isolated monolayers, the heterostructure displays a broader absorption spectrum and a significantly higher absorption intensity in the visible region, with a peak value reaching 2.52 × 10^5^ cm^−1^ at a photon energy of 5 eV, which is comparable with that in other vdW heterostructures, such as GaAs/InS,^34^ PtI_2_/Bi_2_Se_3_,^62^ PtS_2_/g-C_3_N_4_ (ref. 63) and GaAs/Bi_2_Se_3_.^60^ Such an enhanced optical response, together with the efficient charge separation arising from the type-II band alignment and the built-in electric field, indicates that the GaAs/GeH heterostructure is a promising candidate for photocatalytic water splitting applications.

**Fig. 8:**
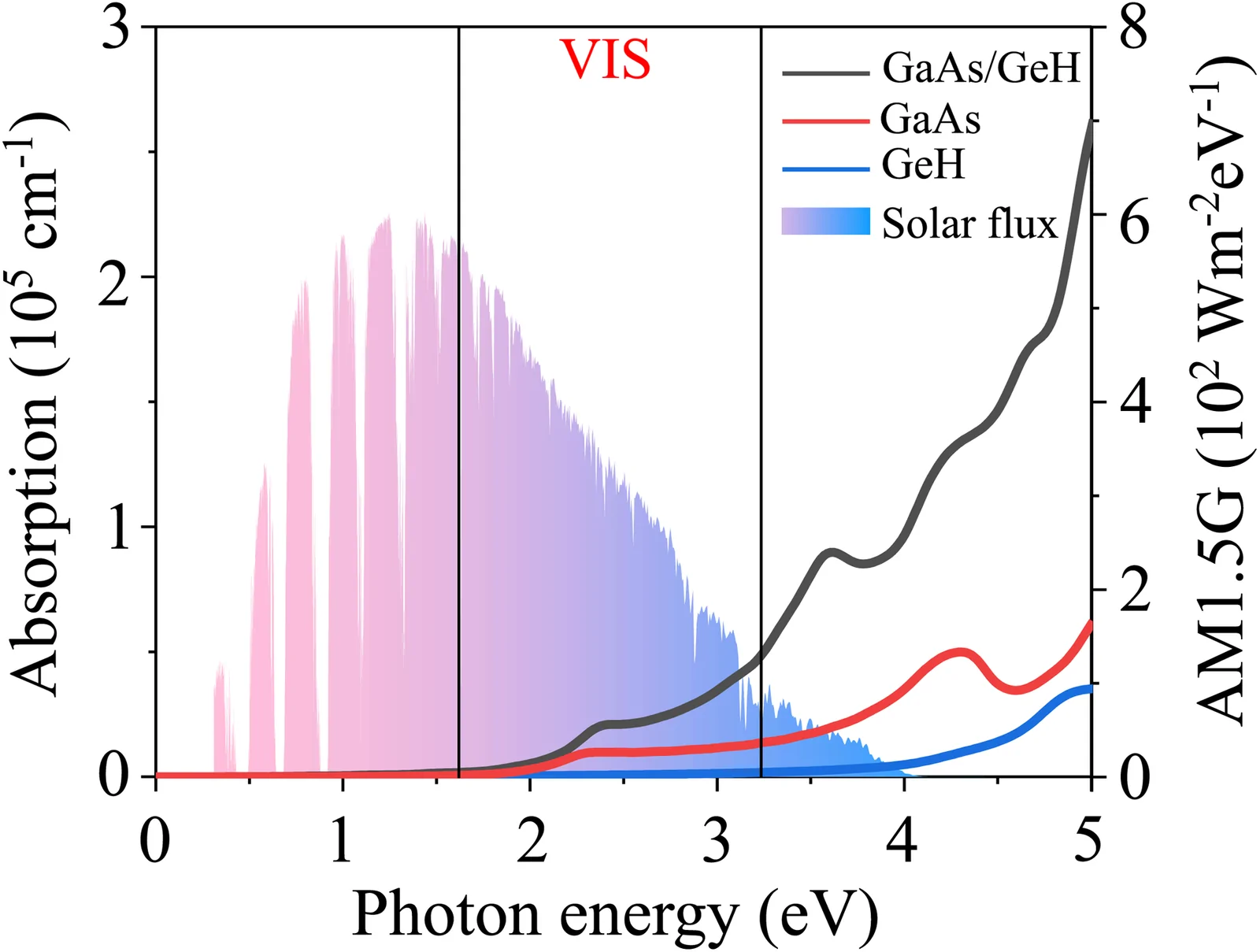
Optical absorption coefficients of the GaAs, GeH monolayers and GaAs/GeH heterostructure.

### 3.8 Photocatalytic properties of the GaAs/GeH heterostructure

To evaluate the suitability of the heterostructure for the HER and OER processes, the absolute positions of the band edges are calculated and depicted in Fig. 9(a). For a photocatalyst to efficiently drive water splitting, its band-edge positions should straddle the standard redox potentials of water, with the CBM located above the hydrogen reduction potential (*E*_H^+^/H_2__ = −4.44 eV) and the VBM positioned below the oxygen evolution potential (*E*_H_2_O/O_2__ = −5.67 eV). It is observed that the CBM of both GaAs and GeH lies above the hydrogen reduction potential, indicating that both materials possess sufficient reduction ability to drive the HER. However, a notable difference appears in the VBM positions. The VBM of the GaAs monolayer is located below the oxygen evolution potential, satisfying the thermodynamic requirement for the OER. In contrast, the VBM of the GeH monolayer is positioned above this level, indicating insufficient oxidizing ability for the OER. Therefore, although both GaAs and GeH possess sufficient reduction capability for the HER, the GaAs monolayer provides the more favorable oxidation capability for the OER.

**Fig. 9:**
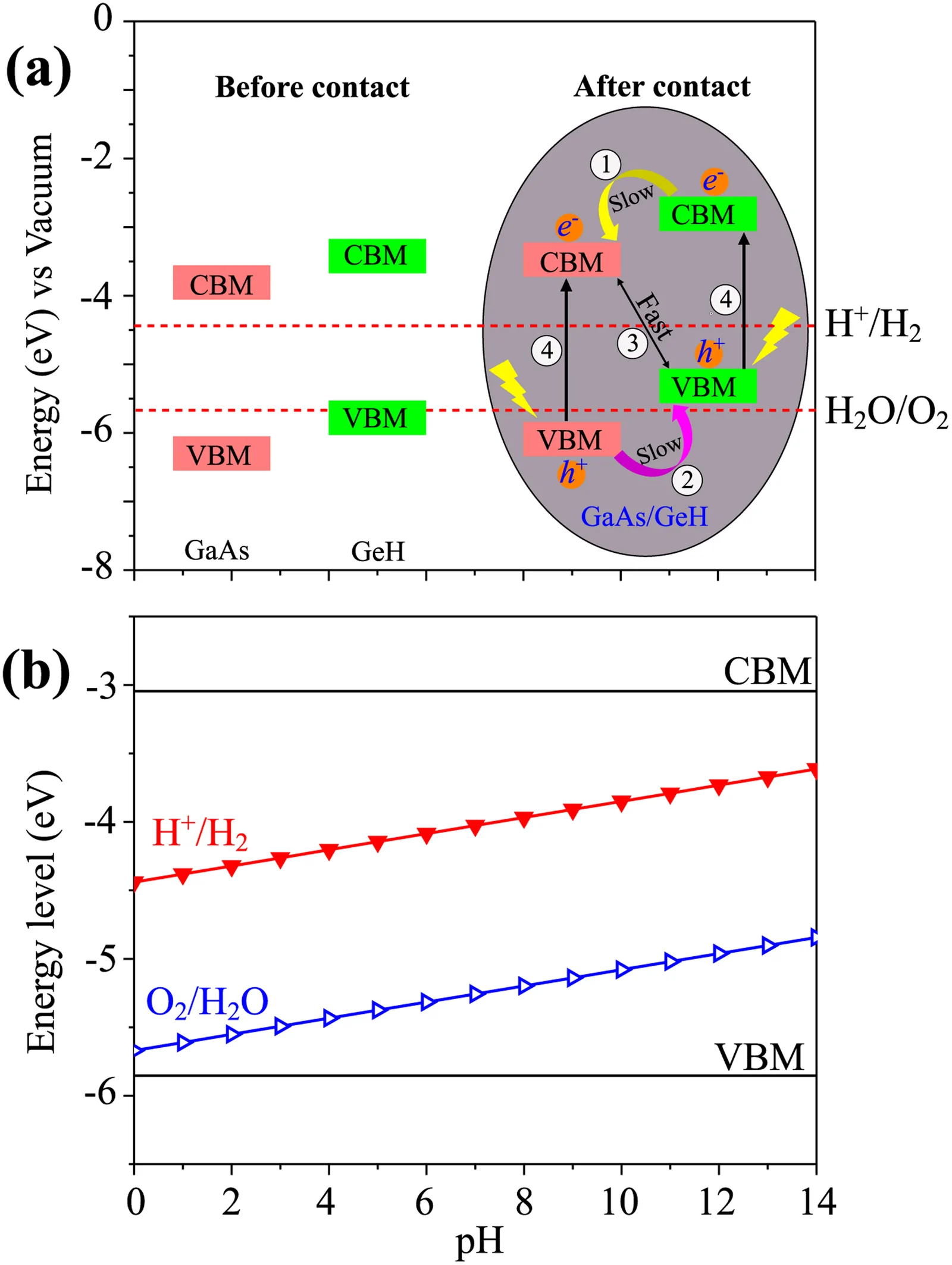
(a) Band alignments of the GaAs, GeH and GaAs/GeH heterostructure and photocatalytic water splitting mechanism at the interface of the GaAs/GeH heterostructures. The symbols of 1, 2, 3 and 4 represent the four distinct migration pathways for charge carriers at the interface of the heterostructure. (b) Band edges of the GaAs/GeH heterostructure with the redox potentials of water splitting as a function of pH.

After formation of the GaAs/GeH heterostructure, the band alignment is modified by the interfacial interaction and charge redistribution. The CBM of the heterostructure remains above the hydrogen reduction potential, indicating that the system retains sufficient reducing capability for the HER. More importantly, the VBM of the GaAs/GeH heterostructure is positioned below the oxygen evolution potential, satisfying the thermodynamic requirement for the OER. Therefore, the heterostructure provides complementary band-edge positions for the two half-reactions, with the GeH-derived CBM providing the reduction capability and the GaAs-derived VBM providing the oxidation capability.

To further elucidate the photocatalytic mechanism of the GaAs/GeH heterostructure, the migration pathways of photogenerated charge carriers are illustrated in Fig. 9(a). Under solar irradiation, electrons in both GaAs and GeH monolayers are excited from the VBM to the CBM (path 4), generating electron–hole pairs. The photogenerated electrons in the CBM of the GaAs layer preferentially recombine with the holes in the VBM of the GeH layer (path 3). Meanwhile, the interfacial electrostatic potential suppresses two unfavorable carrier-transfer pathways, namely, the transfer of electrons from the CBM of GeH to that of GaAs (path 1) and the migration of holes from the VBM of GaAs to that of GeH (path 2). Consequently, electrons are retained in the CBM of the GeH layer, whereas holes remain in the VBM of the GaAs layer. The electrons retained in the GeH's CBM possess sufficient reduction ability to drive the HER, whereas the holes retained in the GaAs's VBM possess sufficient oxidation ability to drive the OER. Overall, the GaAs/GeH heterostructure follows a direct Z-scheme charge-transfer mechanism, in which photogenerated electrons in the GaAs's CBM recombine with photogenerated holes in the GeH's VBM, while the electrons retained in the GeH's CBM and the holes retained in the GaAs's VBM are responsible for the HER and OER, respectively. This selective carrier-transfer pathway preserves the strong redox capability of the photogenerated carriers while reducing unfavorable electron–hole recombination.

Furthermore, the redox potentials associated with the HER (*E*_H^+^/H_2__) and OER (*E*_H_2_O/O_2__) vary with the pH of the solution. This pH dependence can be quantitatively described by the following expression:

6 *E*_H^+^/H_2__ = −4.44 eV + pH × 0.059

and

7 *E*_H_2_O/O_2__ = −5.67 eV + pH × 0.059

The alignment of the band edges of the GaAs/GeH heterostructure with respect to the redox potentials of water splitting as a function of pH is illustrated in Fig. 9(b). It can be observed that the CBM of the GeH layer remains above the hydrogen evolution potential across the entire pH range from 0 to 14, indicating sufficient reducing capability for the HER. Similarly, the VBM of the GaAs layer is consistently located below the oxygen evolution potential under all pH conditions, ensuring adequate oxidizing power to drive the OER. These results demonstrate that the GaAs/GeH heterostructure maintains favorable band-edge alignment with respect to the water redox potentials over a wide pH range. Consequently, the heterostructure is expected to exhibit stable and efficient photocatalytic water splitting performance under both strongly acidic (pH = 0) and strongly alkaline (pH = 14) conditions.

To further evaluate the practical photocatalytic performance of the GaAs/GeH heterostructure, the STH efficiency is calculated as:

8 *η*_STH_ = *η*_abs_ × *η*_cu_

where *η*_abs_ represents the light absorption efficiency and *η*_cu_ denotes the carrier utilization efficiency. The absorption efficiency depends on the band gap of the material, while the carrier utilization efficiency is determined by the alignment of band edges with respect to the water redox potentials. The light absorption efficiency is defined as:9
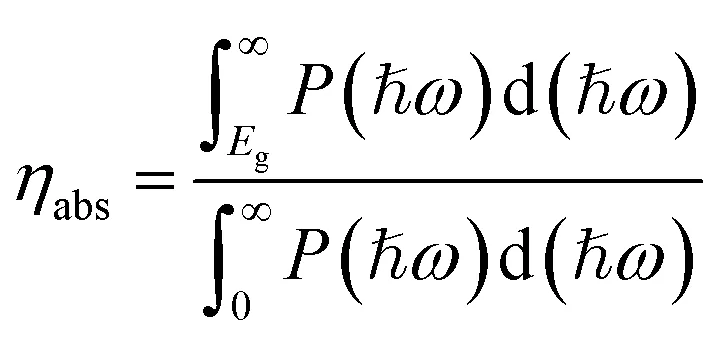
where *E*_g_ is the band gap and *P*(ℏ*ω*) is the solar energy flux. The carrier utilization efficiency is evaluated based on the number of absorbed photons contributing to the water splitting reaction and can be expressed as:10
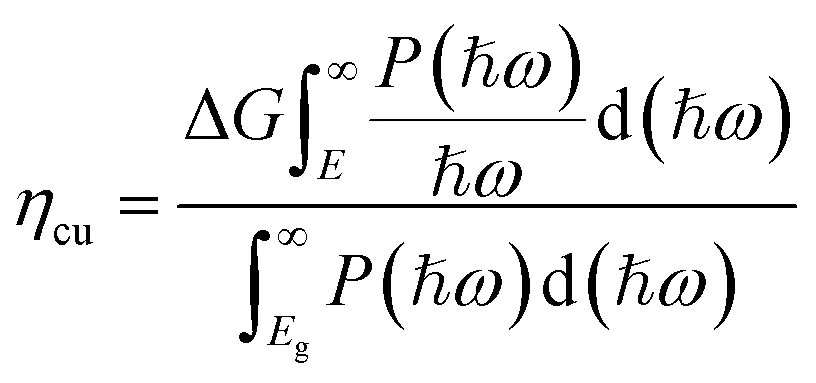
where Δ*G* is the Gibbs free energy required for water splitting. *E* represents the minimum photon energy required for excitation, which is taken to be:11
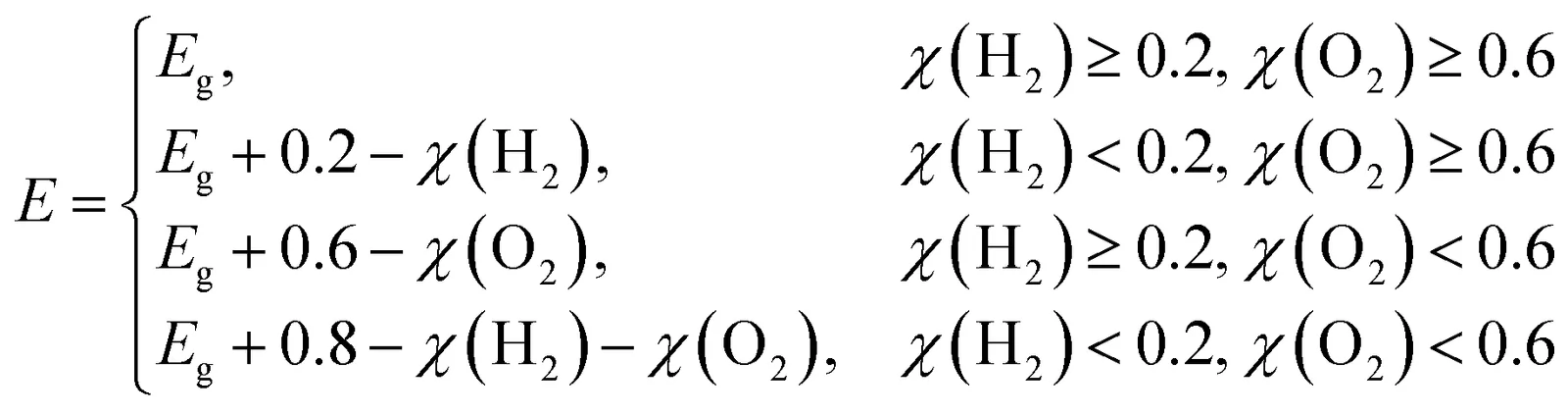
where *χ*(H_2_) and *χ*(O_2_) represent redox driving forces for the HER and OER, respectively, which can be determined as:

12 *χ*(H_2_) = *E*_CBM_ − *E*_H^+^/H_2__

and

13 *χ*(O_2_) = *E*_O_2_/H_2_O_ − *E*_VBM_

It should be noted that, for the GaAs/GeH heterostructure, *χ*(H_2_) and *χ*(O_2_) are evaluated based on the layer-resolved band edges involved in the direct Z-scheme mechanism. Specifically, *χ*(H_2_) is determined from the GeH-derived CBM, whereas *χ*(O_2_) is determined from the GaAs-derived VBM. Therefore, these two quantities do not correspond to the CBM and VBM of a single absorber and should not be used to reconstruct the fundamental band gap of the heterostructure.

The calculated STH conversion efficiencies of the GaAs and GeH monolayers, as well as the GaAs/GeH heterostructure, are summarized in Table 3. The STH efficiency of the GaAs monolayer is found to be 12.42%, which is in good agreement with previously reported values.^33,34,60^ Remarkably, upon forming the GaAs/GeH heterostructure, the STH efficiency is significantly enhanced to 24.41%, which is approximately twice that of the individual monolayers. Furthermore, this value demonstrates a clear advantage over many previously reported type-II heterostructures, such as GaAs/InS (17.06%),^34^ GaAs/Bi_2_Se_3_ (21.83%),^60^ GaAs/GeTe (27.28%),^33^ SiI_2_/InSe (24.31%),^64^ HfSe_2_/InSe (25.24%)^65^ ZnO/Ga_2_SSe (25.05%),^66^ PtSSe/phosphorene (24.86%)^67^ and GeH/InSe (24.72%).^43^ It can be seen that the GaAs/GeH heterostructure exhibits a competitive theoretical STH efficiency of 24.41%, although this value is lower than the 27.28% reported for GaAs/GeTe^33^ and GeH/InSe.^43^ Importantly, the potential of GaAs/GeH is not determined solely by its STH efficiency, but by the synergistic combination of favorable band-edge alignment, a direct Z-scheme photocarrier-transfer pathway, efficient interfacial charge redistribution, strong optical absorption, and favorable mechanical and dynamical stability. These combined characteristics distinguish GaAs/GeH as a promising candidate for solar-driven photocatalytic water splitting.

**Table 3:** The calculated over-potential for the HER (*χ*(H_2_), eV), over-potential for oxygen evolution reaction (*χ*(O_2_), eV), photon energy (*E*, eV), HSE band gap (*E*_g_, eV), light absorption efficiency (*η*_abs_, %), carrier utilization efficiency (*η*_cu_, %) and STH efficiency (*η*_STH_, %) for the GaAs, GeH monolayers, and their GaAs/GeH heterostructure

Materials	*χ*(H_2_)	*χ*(O_2_)	*E*_g_	*E*	*η*_abs_	*η*_cu_	*η*_STH_
GaAs	0.40	0.39	2.02	2.23	35.57	34.92	12.42
GeH	0.78	−0.13	1.88	—	—	—	—
GaAs/GeH	1.40	0.18	1.37	1.79	68.70	35.54	24.41

Finally, we would like to emphasize that the calculated STH efficiency of 24.41% represents an idealized theoretical upper-limit estimate based on the calculated band-edge positions and solar-spectrum absorption. The present estimation does not explicitly account for several practical loss mechanisms, including exciton binding energy, photogenerated carrier recombination, interfacial transport barriers, defect- or trap-assisted carrier losses, and surface reaction kinetics. Therefore, the calculated value should not be interpreted as a directly achievable experimental STH efficiency. A quantitative prediction of the practical STH performance would require explicit consideration of these non-ideal effects and detailed surface reaction kinetics. It should be noted that the present analysis evaluates the thermodynamic feasibility of HER and OER based on the band-edge alignment, rather than the detailed surface reaction kinetics. In particular, explicit calculations of H_2_O adsorption, H adsorption, Δ*G*_H*_, and the OER reaction free-energy pathway were not performed in this work. Such surface-reaction calculations would be required to quantitatively assess the catalytic activity and reaction kinetics, particularly considering the hydrogen-terminated surface of GeH layer. Therefore, the present results should be regarded as evidence for the thermodynamic suitability of the GaAs/GeH heterostructure for photocatalytic water splitting, rather than a complete description of its surface reaction mechanism.

## 4 Conclusions

In this work, a comprehensive first-principles study has been performed to investigate the structural, electronic, mechanical, optical, and photocatalytic properties of the GaAs/GeH vdW heterostructure. The results confirm that the system is structurally stable and exhibits a direct band gap with a type-II band alignment. The formation of the heterostructure leads to a reduced band gap and significantly enhanced optical absorption, particularly in the visible region. In addition, the calculated carrier mobility suggests improved electron transport, which is beneficial for photocatalytic performance. Importantly, interfacial charge redistribution and the resulting built-in electric field promote a direct Z-scheme charge-transfer pathway, preserving the strong redox capabilities required for HER and OER. As a result, a theoretical STH conversion efficiency of 24.41% is achieved, indicating competitive photocatalytic performance. These findings highlight the strong potential of the GaAs/GeH heterostructure for efficient solar-driven water splitting and provide useful insights for the design of advanced 2D photocatalytic heterostructures.

## Conflicts of interest

There are no conflicts to declare.

## Data Availability

The data that support the findings of this study are available from the corresponding author upon reasonable request.
